# A Case of Primary Urethral Malignant Melanoma With Persistent Bleeding and Severe Anemia

**DOI:** 10.1002/iju5.70051

**Published:** 2025-05-25

**Authors:** Tatsuma Juichi, Go Noguchi, Yuki Yokoi, Daisuke Yamashita, Shuko Yoneyama, Kazuhide Makiyama, Akitoshi Takizawa

**Affiliations:** ^1^ Department of Urology International Goodwill Hospital Yokohama Kanagawa Japan; ^2^ Department of Urology Yokohama City University Graduate School of Medicine Yokohama Kanagawa Japan

**Keywords:** mucosal melanoma, urethral malignant melanoma, urethral tumor

## Abstract

**Introduction:**

Primary malignant melanomas of the urethra are extremely rare. The initial symptoms typically include hematuria or genital bleeding. This report describes a case of a massive primary urethral malignant melanoma with persistent bleeding.

**Case Presentation:**

An 84‐year‐old woman presented with bleeding and pain from a vulvar mass and severe anemia. She presented with a fist‐sized dark‐brown multifocal mass extending from the urethra, with persistent bleeding and anemia in the same area. After blood transfusion, tumor resection was performed. Histopathological analysis confirmed the presence of primary malignant urethral melanoma.

**Conclusion:**

Urethral malignant melanoma is a rare and aggressive disease that often presents with symptoms that mimic benign conditions. Despite poor prognosis, early surgical resection with adequate margins is crucial. This case highlights the importance of considering malignant melanomas in the differential diagnosis of atypical urethral masses, particularly in patients with persistent bleeding.

**Summary**

- Primary urethral malignant melanoma is a rare and aggressive tumor that is often misdiagnosed as benign.
- Early diagnosis and surgical intervention with adequate margins are essential, although the prognosis remains poor, with high recurrence rates.
- This case emphasizes the importance of considering malignant melanomas in atypical urethral masses, particularly in patients with persistent bleeding and anemia.

## 1 Introduction

Primary malignant melanoma of the urethra is an extremely rare tumor, accounting for approximately 4% of all malignant urethral tumors [1]. Initial symptoms may include hematuria or genital bleeding [2]. This report presents the case of a massive primary urethral malignant melanoma with persistent bleeding.

## 2 Case Presentation

**FIGURE 1:**
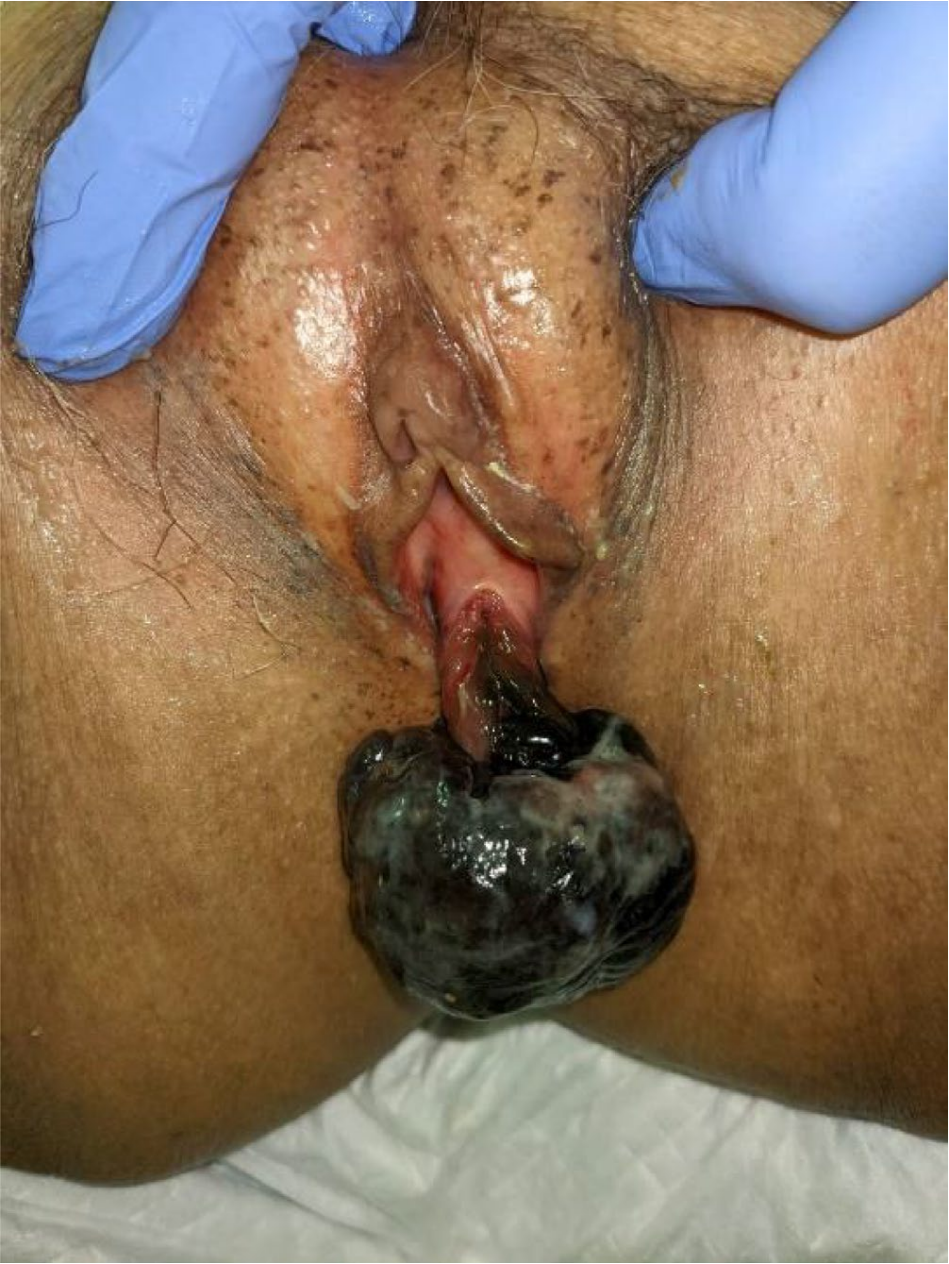
Macroscopic findings: A dark‐brown, multilobular tumor approximately 70 mm in size was observed extending from the 6 o'clock position of the external urethral meatus.

An 84‐year‐old woman with Alzheimer's disease was referred to our hospital for evaluation of genital bleeding, pain, and severe anemia (Hb 6.4 g/dL), which was identified during hospitalization at another institution. Physical examination revealed a dark‐brown multilobulated mass, approximately fist‐sized, originating at the 6 o'clock position of the external urethral meatus, with gradual and persistent venous bleeding and associated pain (Figure 1). No voiding difficulty was observed.

**FIGURE 2:**
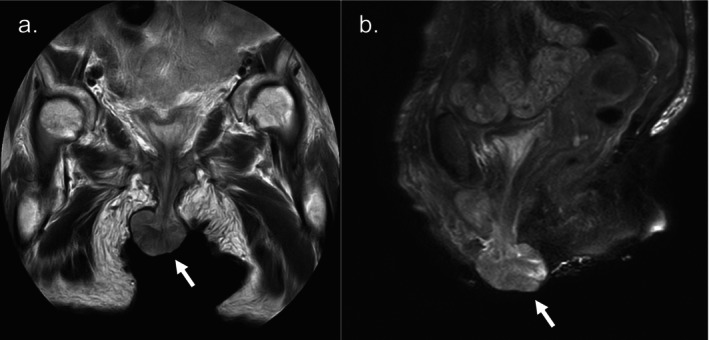
Magnetic Resonance Imaging images: A tumor continuous with the urethra shows mildly high signal intensity on T1‐weighted imaging and high signal intensity with internal striations on T2‐weighted imaging.

**FIGURE 3:**
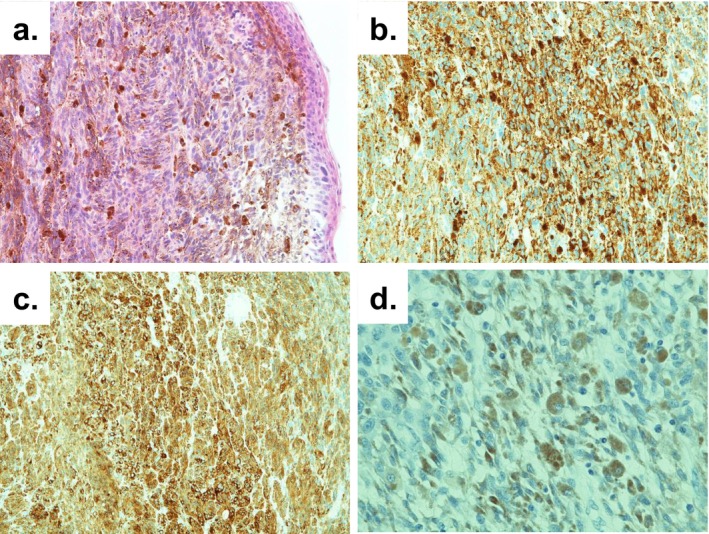
Histological images: (a) Hematoxylin and eosin staining (×200), and immunohistochemical staining showing tumor cells positive for (b) HMB‐45 (×200), (c) Melan‐A (×200), and (d) S‐100 (×400).

Laboratory tests showed no abnormalities except for anemia, and tumor markers (SCC, IL2R, CA19‐9, CEA) and urine cytology were negative. Computed tomography showed no significant abnormalities other than a perineal mass, with no evidence of lymph node or distant metastasis. Magnetic resonance imaging revealed a mass with mildly high signal intensity on T1‐weighted imaging, and high signal intensity with internal striations on T2‐weighted imaging. While no apparent invasion was seen, internal cord‐like structures suggestive of intratumoral hemorrhage were noted. Differential diagnoses included aggressive angiomyxoma and angiomyofibroblastoma (Figure 2). Due to difficulty in achieving hemostasis by compression, an emergency tumor resection was performed following the transfusion of 2 units of red blood cells. Surgery was performed under general anesthesia in the lithotomy position. The mass was manually retracted and excised with as wide a margin as possible, centered on the 6 o'clock position of the urethral meatus. After securing hemostasis, the resection site was ligated and sutured with Vicryl. Cystoscopy was then performed, confirming no residual tumor or bleeding in the urethra or bladder. The operation took approximately 30 min, and blood loss was minimal. The postoperative course was uneventful, with no further bleeding, progression of anemia, or urinary dysfunction. The resected specimen was a solid, dark‐brown tumor measuring 50 × 45 × 30 mm. The cut surface was solid, without evidence of hematoma. Histopathological examination revealed atypical cells forming a solid mass with prominent nucleoli. Histopathological examination revealed atypical cells with round and prominent nucleoli forming a solid mass. Immunohistochemical staining revealed HMB45(+), melan‐A (+), S100(+), and AE1/AE3(−), leading to a diagnosis of malignant melanoma (Figure 3). Due to tissue crush artifact, resection margins and depth of invasion could not be accurately assessed, and mucosal infiltration could not be excluded. No other cutaneous or mucosal melanoma lesions were identified, suggesting primary urethral origin. Although additional treatment such as cystourethrectomy was considered, both the patient and her family opted for observation. At 1 year and 9 months postoperatively, a 5‐mm mass reappeared at the 6 o'clock position of the urethral meatus, raising suspicion of local recurrence. No bleeding or distant metastasis was observed. Considering the patient's advanced age and cognitive decline, the patient and her family chose best supportive care without further biopsy or excision, and ongoing observation was continued.

## 3 Discussion

Urethral malignant melanoma is classified as a mucosal melanoma. Mucosal melanoma accounts for approximately 1.3% of melanomas and can arise in any mucosal epithelium containing melanocytes [3]. Of these, melanomas of the urinary tract represent only 2.8% [4], making urethral malignant melanoma an extremely rare disease. The average age of onset is 64 years, and it occurs three times more frequently in women than in men [2]. Reported symptoms include a palpable mass, urinary retention, bleeding, hematuria, decreased urine stream, incontinence, pain, and weight loss, although some cases are asymptomatic [5]. Because these tumors often arise at the urethral meatus or distal urethra, [2] they are frequently misdiagnosed as urethral polyps or caruncles [6]. Preoperative diagnosis of malignant melanoma is achieved in only 5.2% of cases [7].

The prognosis of urethral malignant melanoma is extremely poor. At the time of diagnosis, 50% of patients already have metastases, and the reported mean survival time is 25.6 months [2]. Complete urethrectomy at an early stage has been reported to reduce the risk of local recurrence; [8] however, even with surgical intervention, recurrence rates within the first year are approximately 70%, [2] making tailored surgical approaches based on the patient's condition essential. Recently, various immunotherapies have been explored with the expectation of improved survival in patients with urethral malignant melanoma [9].

We report a rare case of primary urethral malignant melanoma presenting with persistent bleeding and severe anemia. The tumor measured 50 mm in diameter, which is larger than the previously reported average of 2.2 cm [2]. The delay in consultation, possibly due to the patient's underlying Alzheimer's disease, may have contributed to the tumor's growth. Malignant melanomas with hemorrhage are often misdiagnosed as hematomas [10]. In this case, the tumor was also initially presumed to be largely hematomatous, and resection was performed without a prior biopsy for the purpose of hemostasis. However, pathological analysis revealed that the dark‐colored areas consisted entirely of tumor cells. The anemia was thought to result from continuous bleeding from the expanding tumor surface.

In nonmetastatic cases, surgical resection with at least a 2‐cm margin is recommended for curative treatment [11]. In our case, because melanoma was not suspected preoperatively, we resected the tumor including the urethral mucosa at its base, but without securing a 2‐cm margin. Furthermore, the urethral margin was not marked at the time of specimen fixation, making accurate pathological evaluation of the depth of invasion and surgical margin status impossible. This remains a critical point for reflection. When resecting urethral tumors, the possibility of malignancy should always be considered, and proper marking of the urethral margin prior to fixation is essential.

Therefore, when encountering an atypical hemorrhagic tumor in the urethra, the possibility of malignant melanoma should be included in the differential diagnosis, and wide excision with clearly defined surgical margins is strongly recommended.

## 4 Conclusion

We encountered a rare case of a massive primary urethral malignant melanoma with persistent bleeding and severe anemia. When an atypical urethral tumor is identified, malignant melanoma should be considered, and surgical excision with an adequate margin is essential. Additionally, proper marking of the urethral margin during specimen fixation is crucial to enable accurate pathological assessment of surgical margins and depth of invasion.

## Consent

Informed consent was obtained from the patient and her family for the publication of this case report.

## Conflicts of Interest

The authors declare no conflicts of interest.
